# Supplementary material for: Vision-driven metasurfaces for perception enhancement
Source: Nat Commun. 2024 Feb 22;15:1631. doi: 10.1038/s41467-024-45296-x (PMC10883922; doi:10.1038/s41467-024-45296-x)
Supplement: Supplementary file 1 — Supplementary Information [file 41467_2024_45296_MOESM1_ESM.docx]

**Supplementary Note S1: Design of the unit cell and the metasurface**

The metasurface consists of top layer and ground layer, which are spaced by two layers of polyethylene terephthalate (PET) substrates with a height *h* = 0.125 mm and one layer of polycarbonate (PC) with a height *h* = 0.8 mm, respectively, as illustrated in Fig. S1. As optically transparent substrates, PC layer is used to support and isolate the metallic patterns while PET is used for the etching of copper. PC and PET are bonded by UV-curable adhesives to meet the requirements of light transmittance and processing temperature.


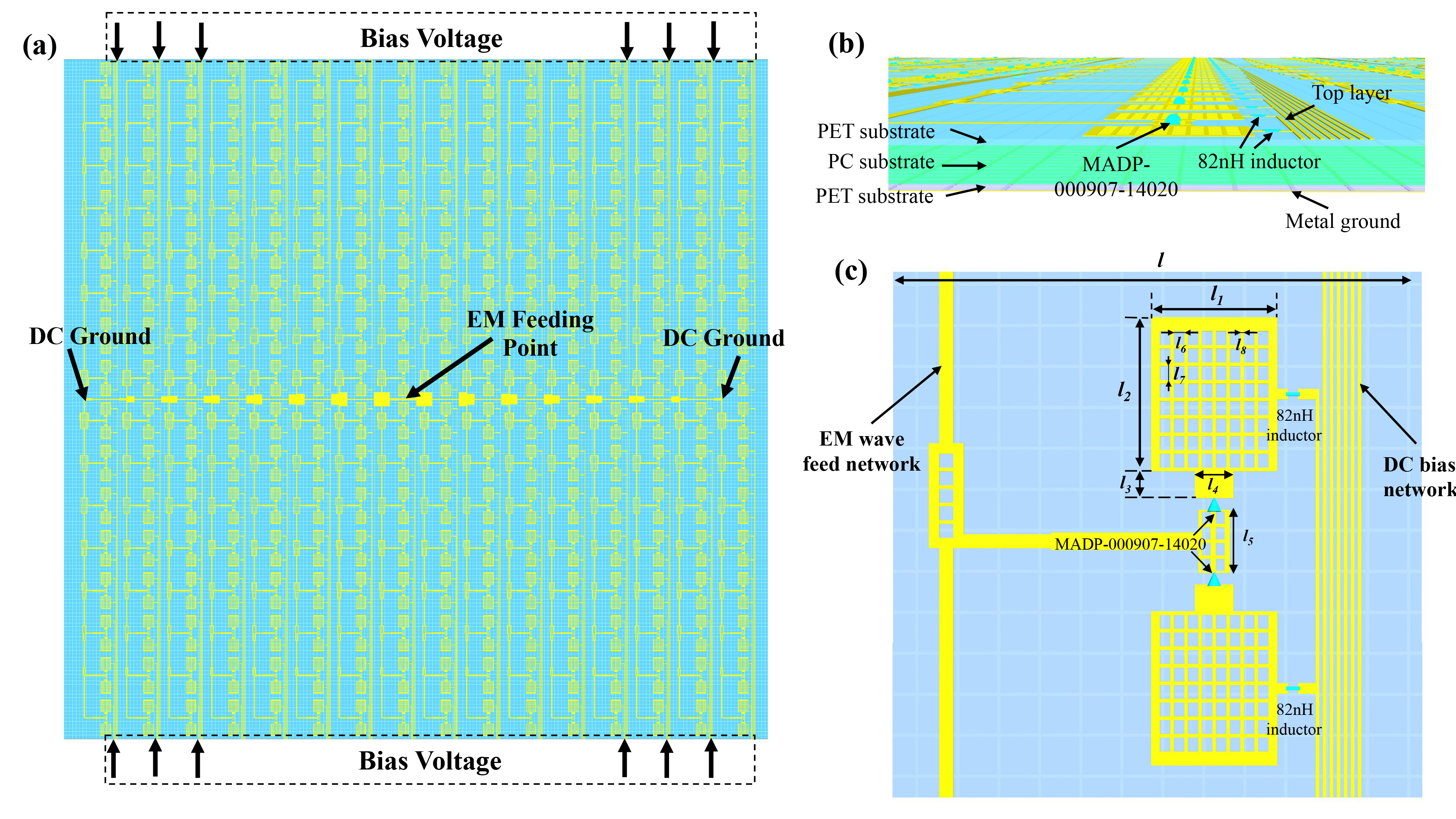


Fig. S1. Overall configuration of the proposed radiation-type metasurface with 16 × 16 units. (a) perspective view and (b) lateral view. (c) Metasurface unit cell topology, the dimensions of the unit cell are *l* = 15 mm, *l_1_* = 3.6 mm, *l_2_* = 4.4 mm, *l_3_* = 0.8 mm, *l_4_* = 1.1 mm, *l_5_* = 1.8 mm, *l_6_* = 0.3 mm, *l_7_* = 0.4 mm and *l_8_* = 0.1 mm.

The metal meshes etched on the bottom layer act as the metal ground, a 1.2 mm mesh spacing and a 0.1 mm wire width are selected in consideration of optical transparency and the minimum wire width processing limit. The metal pattern etched on the top layer consists of an integrated EM wave feed network, periodic unit cells and a DC bias network. There are 16 × 16 periodic cells on the top layer, each consisting of two rectangular copper meshes bridged to the EM wave feed network by two PIN diodes. The PIN diodes anti-symmetrically integrated in unit cells are MADP-000907-14020 from MACOM Technology. The DC bias network supplies DC voltage to the diodes integrated on the metasurface units. The entire DC feed circuit loop is set up by sharing part of the network with the integrated EM wave feed network. A total of 512 PIN diodes are individually controlled by the steering-logic board via 256 independent narrow-width (0.1 mm) bias lines. Chip inductors of 82 nH are used in the DC feed circuit loop to minimize the impact of the DC bias network on the metasurface’s radiation performance and ensure a flat gain response. The EM wave is stimulated at the center of the top layer and propagates through the integrated EM wave feed network which introduces an initial amplitude and phase distribution. Impedance matching is used at the connection between the feed network and unit cell to improve operation bandwidth and loss efficiency. The metasurface is designed to operate in 14.5 GHz with a size of 240*240 mm^2^. Fig. S2(a) shows the fabricated metasurface sample and Fig. S2(b) shows a contrast of photos taken through/without metasurfaces. The effect of observing an object through the metasurface is similar to that of observing an object through a screen window, which ensures optical viewing through the metasurface.


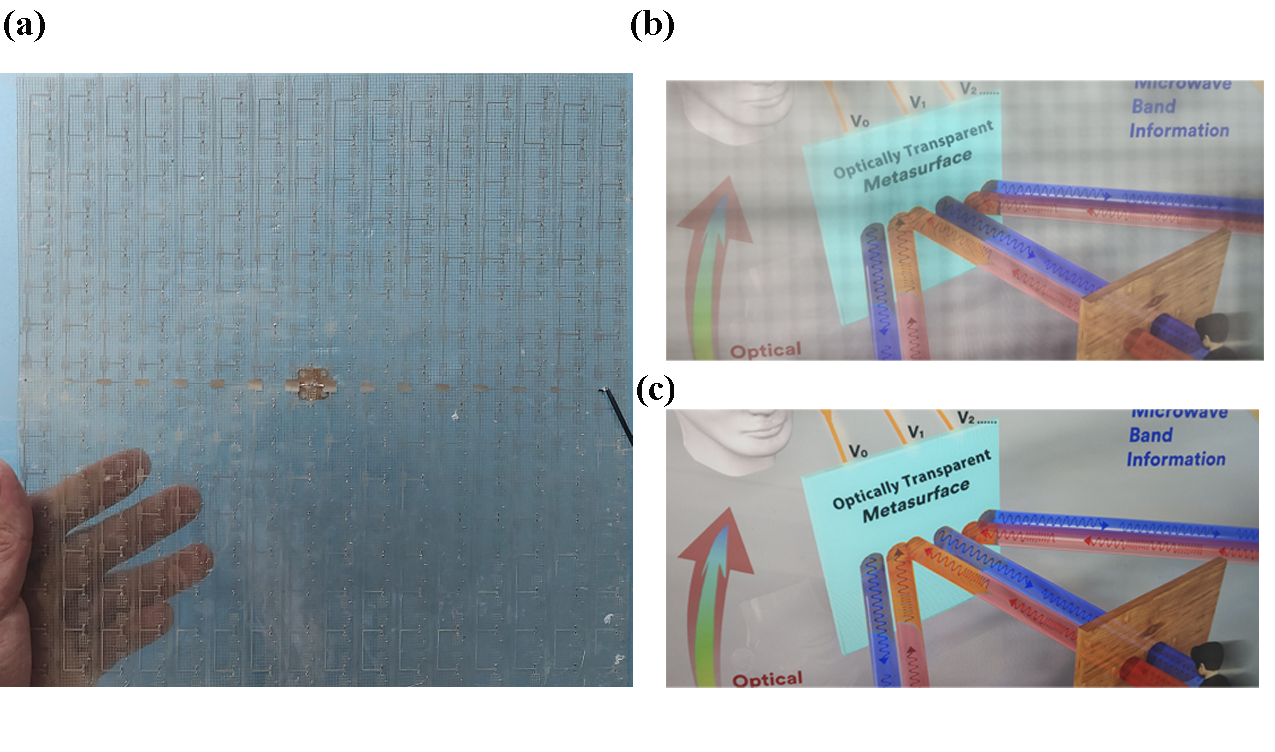


Fig. S2. (a) Photographs of the fabricated prototype and (b-c) contrast of photographs taken through/without metasurfaces.

**Supplementary Note S2: The equivalent RLC circuit** **of PIN diodes**

It is well known that the analysis of the electromagnetic (EM) response of metasurface unit cells typically relies on iterative numerical calculations. In this note, an equivalent circuit model of PIN diodes is used instead of the SPICE model in CST simulation to speed up this procedure. To obtain the equivalent parameters of the RLC, we measured the *S*-parameter of MADP-000907-14020 under different DC bias. Effective circuit parameters of the RLC series circuit are turned to fit with the *S*-parameter in Advanced Design System software. In this way, the effective circuit parameters of MADP-000907-14020 are extracted.

Fig. S3 shows the effective RLC series resonant circuit of MADP-000907-14020 for forward bias and reverse bias, respectively. In simulations, the PIN diode is modeled as lumped resistance with the typical value of R = 7.8 Ω and an inductance of L = 30 pH for forward bias (“ON” state), whereas for reverse bias (“OFF” state), it acts as a lumped capacitance with the typical value of C = 0.025 pF and an inductance of L = 30 pH.


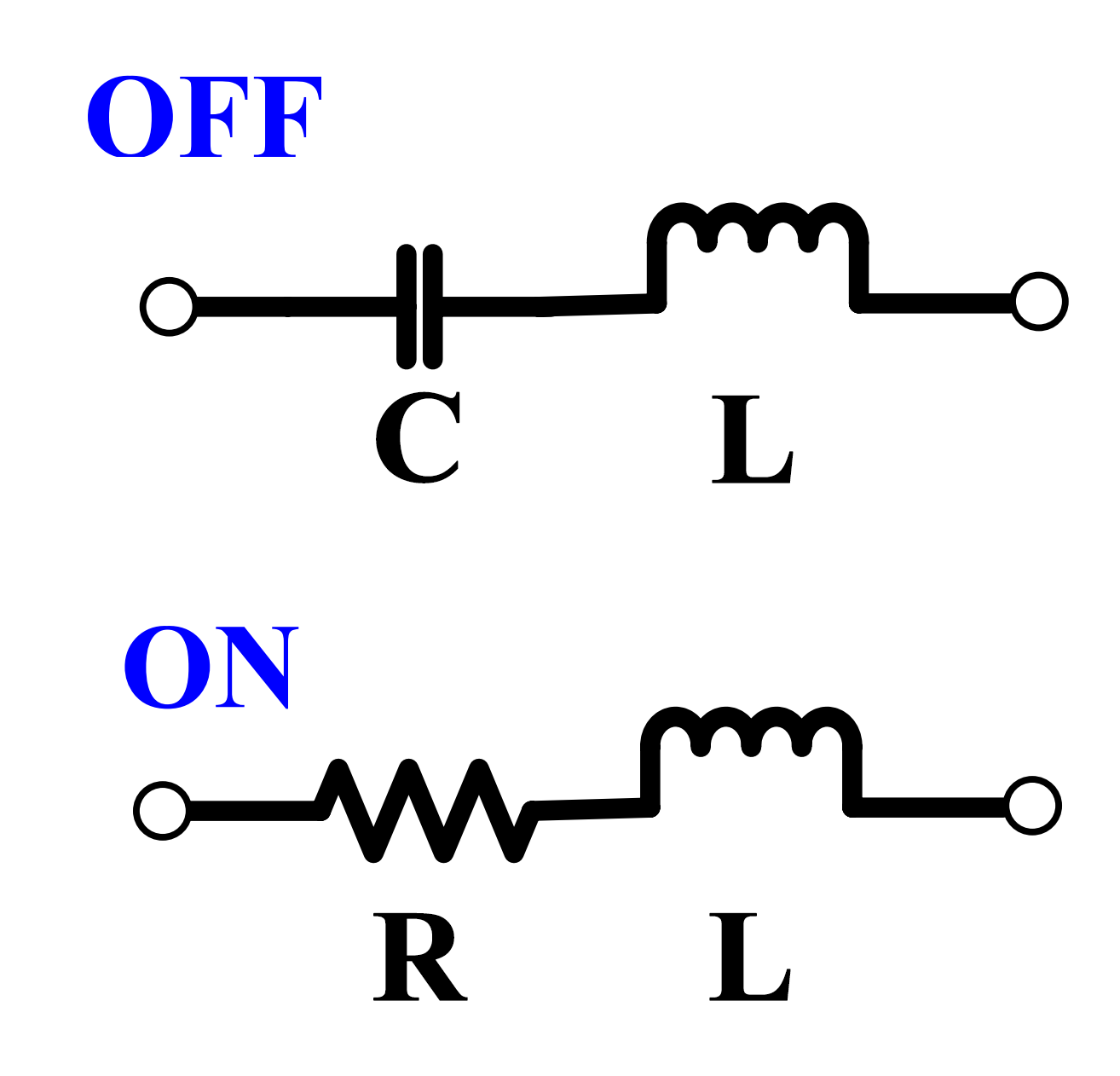


Fig. S3. The equivalent circuits of the PIN diode (MACOM MADP-000907-14020) at "ON" and "OFF" states, respectively.

**Supplementary Note S3: The principle and method of eye movement recognition**

Eye tracking has long been known and used as a method to study the visual attention of individuals. There are several different techniques to detect and track the movements of the eyes. For the remote non-invasive eye tracking scenario in this paper, Pupil center corneal reflection (Pupil-CR) is adopted. The basic concept is to use a near-infrared light source to illuminate the center of the eyes (pupil), causing detectable reflections in both the pupil and the cornea (the outer-most optical element of the eye). These reflections (the vector between the cornea and the pupil) are tracked by an infrared camera. The image captured by the camera is then used to identify the reflection of the light source on the cornea (glint) and in the pupil. We can calculate a vector formed by the angle between the cornea and pupil reflections—the direction of this vector, combined with other geometrical features of the reflections, is then used to calculate the gaze direction. The eye movement system (7invensun Asee glasses) is compliance with Low Voltage Directive (LVD) EN 62471:2008, which proves that the detection does not cause physical harm to the eyes, retina, vision and skin.

The collection and extraction of eye movement information mainly relies on the cooperation of the eye-track hardware and the software. The hardware part of the eye tracker is responsible for collecting the eye movement information of the test subject. While the software part converts eye movement information into corresponding control semantics. On this basis, we apply real-time eye movement data and extract the required real-time eye movement information or image information to control and guide the functions of metasurface.


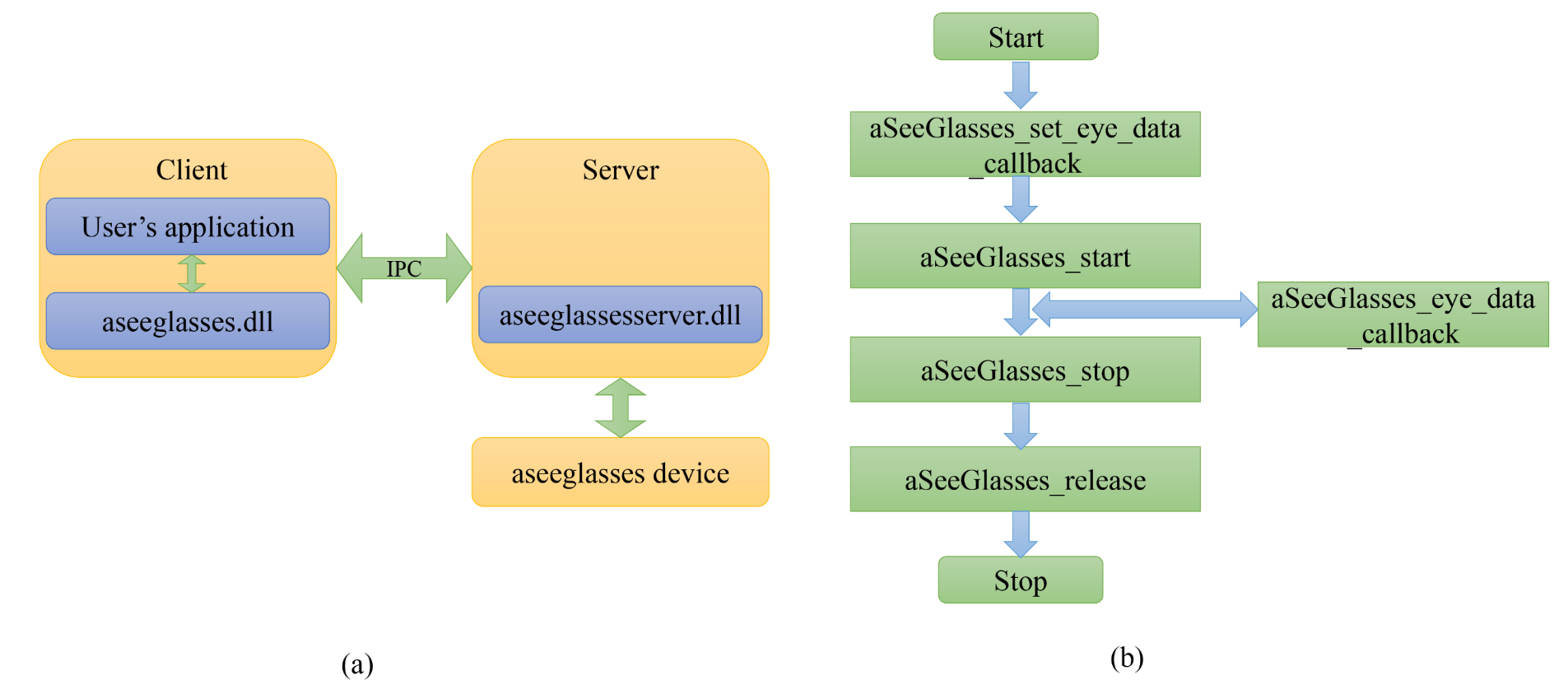


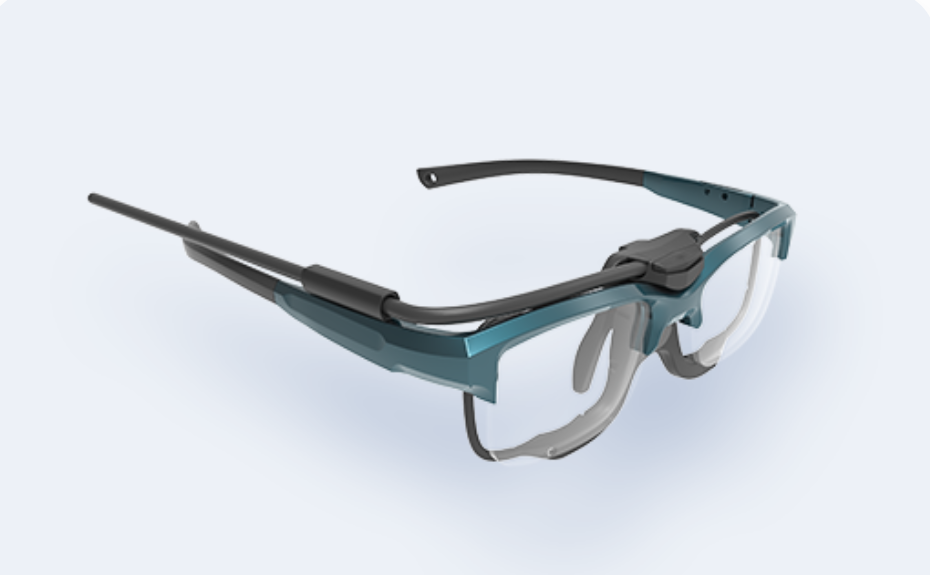


(c)

Fig. S4. (a) The detailed deployment architecture diagram and (b) the flow chart of gaze points tracking process and (c) photograph of adopted eye tracker.

In experiment, the eye tracker is conducted using ASee glasses eye movement system from 7invensun. The photos of eye tracker, detailed deployment architecture and flow chart of gaze points tracking process are shown in Fig.S3. The eye-tracker, connected to the server, transmits collected image information to the server. Through eye tracking algorithm, the eye movement information including gaze point data are obtained. The user needs to set up the service through client (aseeglasses.dll) and communicate with server (aseeglassesserver.dll) by calling the API provided with aSeeSlassesServer.dll to control the eye tracker. In this way, the information interaction can be completed, as shown in S4(a). The flow chart of gaze points tracking process is shown in Fig.S4(b). After the above steps are completed, the user wears the eye tracker and runs the program, and the relevant eye movement data such as the coordinates of the user's viewpoint can be extracted in real time.

**Supplementary Note S4: The experimental configuration for autonomous beam control via** **eye movements**

The experiment aimed to measure the far-field radiation of the prototype under different eye movements. The experimental process was performed in an anechoic chamber for convenience of obtaining accurate results and eliminating interference. The experiment has two major modules, eye track measurement and far-field measurement. Fig. S5 illustrates the autonomous beam control measurement experimental setup.


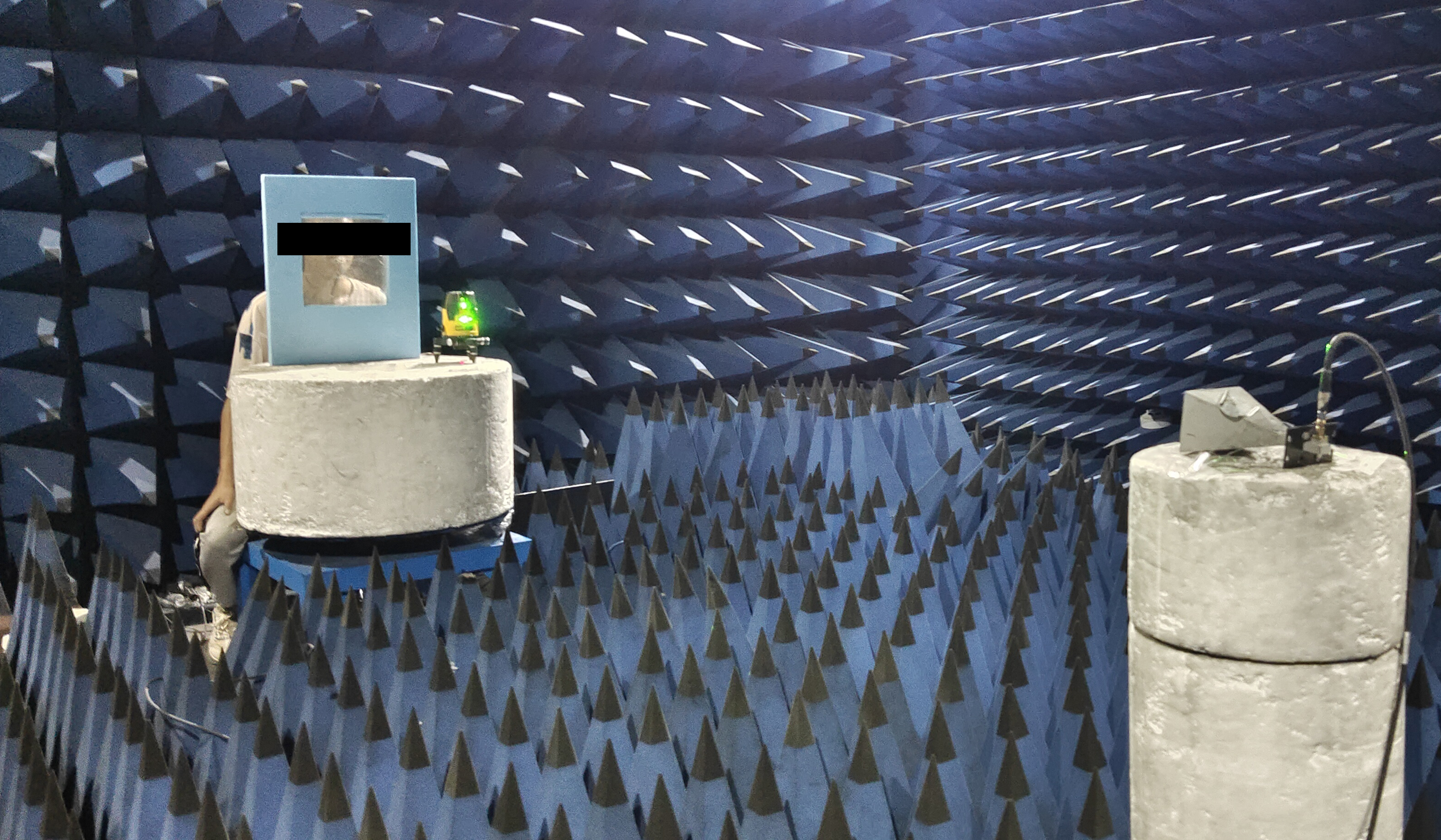


Fig. S5. Experimental setup for the far-field radiation measurement.

In eye track measurement, the observer with normal vision was behind metasurface and wore eye tracking glasses. Next, the observer was asked to keep their heads as still as possible during the experiment and then continuously fixate the specified direction which had been marked by laser over the time threshold. At this time, the radiation beam was turned to the angle corresponding to the angle of sight. On this basis, we experimentally measured the far-field radiation pattern of the coding metasurface under different eye movements, that is, different coding sequences. During the measurement, the EM radiation direction of the metasurface remains the same. The EM measurement system consisted of one horn antenna, a bracket, a rotary platform, and a Keysight N5224A vector network analyzer. Horn antenna connected with the vector network analyzer is chosen as receiving antenna, which is the standard-gain antenna used for receiving linearly polarized waves. The rotation degrees of rotary platform are restricted from -90 to 90˚ to ensure the optimal observation area of the observer. The measured scattering far-field patterns at 14.5 GHz can be obtained through the 180˚ rotation of the rotary platform, where the bracket was placed.

**Supplementary Note S5: The theory of respiration and heartbeat detection**

Unlike the traditional contact‐based vital signs monitoring systems, the proposed metasurface based respiration and heartbeat signal detection system does not require any contact sensors to be attached to the human body. Instead, it relies on the human body’s modulation of an incident EM signal emitted by the metasurface platform. The metasurface radiates a single‐tone narrow beam towards the chest of human subject. The chest‐wall displacement of human subject, which is caused by the respiration and heartbeat would modulate the incident EM wave. This information would be collected by the receiving antenna. After demodulating and processing the received signal, accurate respiration and heartbeat signal can be obtained. The measured peak surface of heartbeat signal corresponds to the maximum mechanical deformation (outward) at the end of the diastolic period, while the trough corresponds to the mechanical deformation (inward) at the end of the systolic period. A complete period of the heartbeat signal measured by metasurface is approximately the period of a sine wave, which is the period of the deformation of the body surface corresponding to a cardiac cycle. In other words, the period from the start of one heartbeat to the start of the next.

The transmitted time‐domain signal *s_T_*(*t*) and the received signal *s_R_*(*t*) can be denoted as follows,

$s_{T}\left( t \right)=A_{t}\cos\left( \omega t \right)$ (S1)

$s_{R}(t)=A_{r}\cos\left[ \omega t-\frac{4\pi}{\lambda}\left( d_{0}+x\left( t \right) \right) \right)]$ (S2)

where *A_t_* and *A_r_* are the amplitudes of the transmitted and received signals, respectively; *ω* is the angular frequency of the transmitted signal, *λ* is the wavelength of carrier frequency, *c* is the speed of light, *d_0_* is the constant distance between the metasurface platform and the human subject, *x*(*t*) is the instantaneous chest-wall displacement, given by,

$x\left( t \right)=A_{b}\cos\left( \omega_{b}t+\varphi_{b} \right)+A_{h}\cos\left( \omega_{h}t+\varphi_{h} \right)$ (S3)

Where *A_b_*, *A_h_*, *φ_h_* and *φ_b_* are the amplitudes and the phase shifts of the chest-wall displacement caused by breathing and heartbeat, respectively. As clearly seen in equation S1 and S2, the signal radiated by the metasurface platform is modulated in both frequency and phase due to the displacement of the chest wall. This modulation is called the Doppler modulation.

The in‐phase baseband signal$s_{I}\left( t \right)$ is obtained by mixing a replica of the transmitted signal in S1 with the received signal in S2,

$s_{I} \left( t \right)=s_{T}\left( t \right)\times s_{R}(t)$ (S4)

Similarly, the quadrature baseband signal is obtained by mixing the received signal with a replica of the transmitted signal shifted by a phase of 90°,

$s_{Q} \left( t \right)=s_{T}\left( t-\frac{\pi/2}{\omega} \right)\times s_{R}(t)$ (S5)

The above *I* and *Q* baseband signals are then combined and used as input in the subsequent signal processing process to determine the respiration and heartbeat signals. With the baseband signal available at hand, to accurately extract the vital signs, we employ the most commonly utilized arctangent demodulation (AD) technique. A general block diagram of the algorithm is shown in Fig. S6.

The detailed steps for the implementation of the algorithm are as follows: The quadrature baseband signal is first divided by the in‐phase baseband signal, and the arctangent of the resulting signal is calculated. In this way, the phase variation information along with the time is extracted, which is actually the desired chest-wall vibration signal. Secondly, a phase unwrapping procedure is applied to the extracted phase signal. Because the phase value is between [-π, π], we need to unwrap it to get the actual displacement of the chest wall. This is realized by performing subtraction of 2π from the phase wherever the phase difference between continuous values is greater than/less than ±π. Thirdly, a phase difference operation is performed on the unwrapped phase to enhance the heartbeat signal and eliminate any potential phase shifts. Then, according to the difference of respiration and heartbeat frequency, bandpass filtering is designed and applied to filter the phase signal to separate the respiration signal and the heartbeat signal. Finally, fast Fourier transform (FFT) is performed to obtain the corresponding respiration frequency.


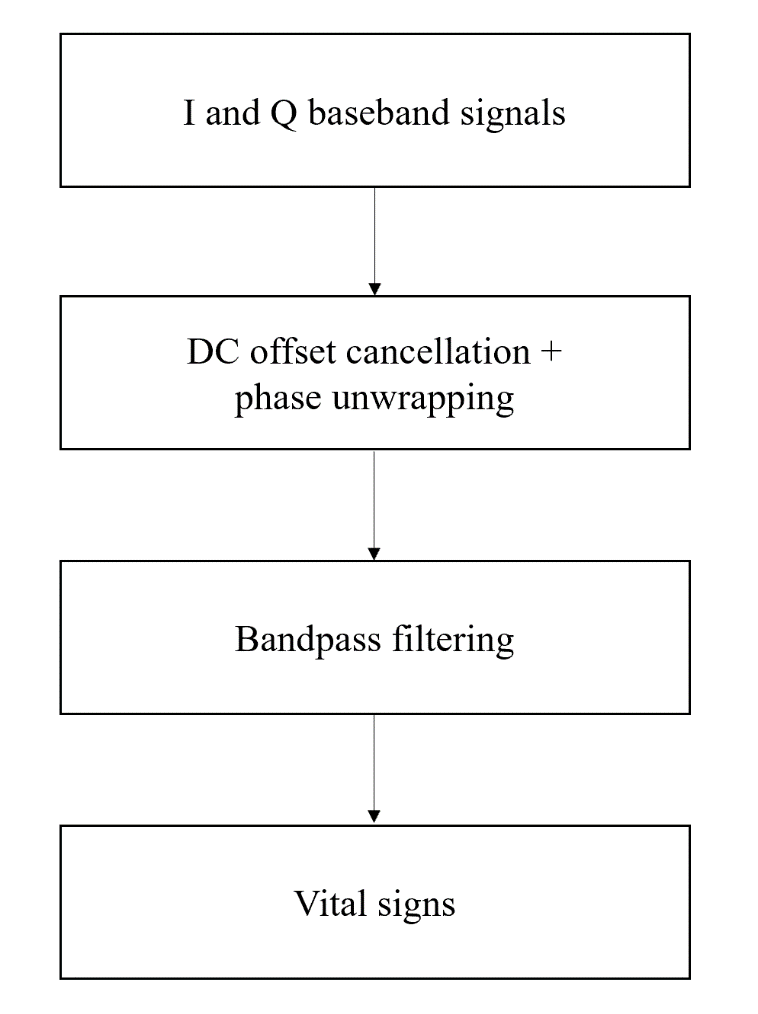


Fig. S6. Flow chart of arctangent demodulatic.

For the filtering procedure, we implement the finite impulse response (FIR) bandpass filtering. A fourth-order FIR bandpass filter with a passband from 0.1 Hz to 0.5 Hz is designed to filter the respiration signal, and another fourth-order FIR bandpass filter with a passband from 0.8 Hz to 2 Hz is designed to filter the heartbeat signal. By exploiting the aforementioned filtering operation, the heartbeat signal can be separated from the respiration signal. And from our experiment results, it is observed that the separated respiration and heartbeat signals align perfectly with the reference signals.

**Supplementary Note S6: The theory and** **algorithm of human location as well as motion detection behind plank obstacles**

1. **Through plank human targets localization using vital signs**

In practical applications such as urban security, the operator often wants to probe the knowledge of hidden space before entering it. Through plank or through wall detection becomes a necessity. Traditional radar-based approach typically relies on large antenna arrays to form a perception aperture and thus restore the imagery of hidden space by utilizing various focusing algorithms. However, since the hidden space is typically with complex distribution of indoor personnel and interference, which cannot be accessed in advance, strong clutters would appear in the imaging result, which can often overwhelm the weak human subjects. It thus makes accurate localization of human targets difficult.

With the vital signs detection method available at hand, through plank human target detection can be easily realized by switching the directions of the narrow beam radiated by metasurface platform. As long as the respiration signal is detected in a specific direction, it can be concluded that a human target presents in that azimuth. In this section, the localization of human personnel is achieved by extracting the respiration signal, which method has been detailed in Supplementary Note S5. Please kindly refer to that section.

1. **Through plank human motion recognition using PCA-SVM**

Microwave based human motion detection has been intensively studied due to its high sensitivity to small movements and excellent ability to distinguish non-stationary objects from a stationary background. The periodic movement of the body torso and limbs of a human subject will introduce micro-Doppler shift to the backscattered EM echoes. This information characterizes as micro-Doppler signature that can be utilized to discriminate different human motions. The metasurface platform emits a directional narrow beam to illuminate the human target in motions, while the receiving antenna collected the reflected signals from the human. The emitted signal is a single frequency continuous wave signal, with the same expression as in equation S1).

The backscattered signal, which contains rich modulated human motion information, can then be expressed as,

$s_{B}(t)=A_{b}\text{exp}\left[ -j2\pi f_{0}\frac{2(R-v_{b}t)}{c} \right]$ (S6)

where *R*-*v_b_t* denotes the real-time human body parts’ location at time instance *t*. After obtaining the baseband signal, its phase is extracted as a function of time. Then, the Doppler frequency shifts induced by the moving body parts being illuminated by the narrow beam is given by,

$f_{b}=\frac{1}{2\pi}\frac{d\varphi}{dt}=\frac{2v}{\lambda}$ (S7)

The two components are sampled at a sampling rate of *f_s_* = 500 Hz Based on the sampling rate, the resulting spectrogram will present a maximum Doppler frequency shift of *f_s_*/2 = 250 Hz, which frequency range is large enough to retain the significant micro-Doppler frequency components of human body parts in motion. The collected *I* and *Q* channel data are then combined to generate the time-domain complex signal, which contains the micro-Doppler information corresponding to the unique movements of human target.

In order to capture the transient behavior of micro-Doppler components, the joint time-frequency information, which provides significantly meaningful feature representation of human motions than the raw time domain signals, is extracted from the received signal using the short-time Fourier transform (STFT),

$X(t,f)=\sum_{\tau=-\infty}^{+\infty} x\left( \tau\right)h(\tau-t)e^{-i2\pi f\tau}d\tau$ (S8)

where *x*(*τ*) is the time-domain samples of the collected signal, and *h*(*τ-t*) is a windowing function that restricts the signal to a short-time frame of a fixed duration of samples. We utilize a Hamming windowing function that has soft edges in this work in order to reduce the effect of spectral leakage caused by the sharp edges of the window. The spectrogram is obtained by sliding the window frame over the entire duration of the captured signal, and calculating the squared amplitude spectrum for each frame.

The length of the Hamming window is *T_w_* = 250 ms, which gives *N* = *f_s_* ×*T_w_* = 256 samples. Each STFT window produces N frequency points. The width of the Hamming window is selected as a trade-off between the time resolution and the frequency domain. The overlap between adjacent window frames is set to be *α* = 90%, which is equal to 230 sample points.

After the motion dataset is collected and processed using the STFT method, we continue to investigate the motion recognition performance for different human motions. Typically, motion detection includes two key phases, feature extraction and classification. In this work, at the first stage, the principal component analysis (PCA) based dimensionality reduction method is employed to extract characteristic motion features. Then, in the classification stage, the support vector machine (SVM) classifier is employed to discriminate different motions. The key concept of SVM is to find a hyper-plane that creates a boundary between the types of data and determine the label of each test sample based on the trained model.

For the motion classification in this work, we utilize the raw spectrograms as inputs to SVM classifier. The dataset is composed of the aforementioned 4 classes of motions. For each motion, we have a total number of 400 samples. 80% of the dataset is utilized to train the classifier, while the rest is utilized for testing. The training and testing sets are selected randomly. Based on the observation, 20 dominant principal components are employed for the classification.

**Supplementary Note S7: The experimental implementation details for "X-ray Glasses"**


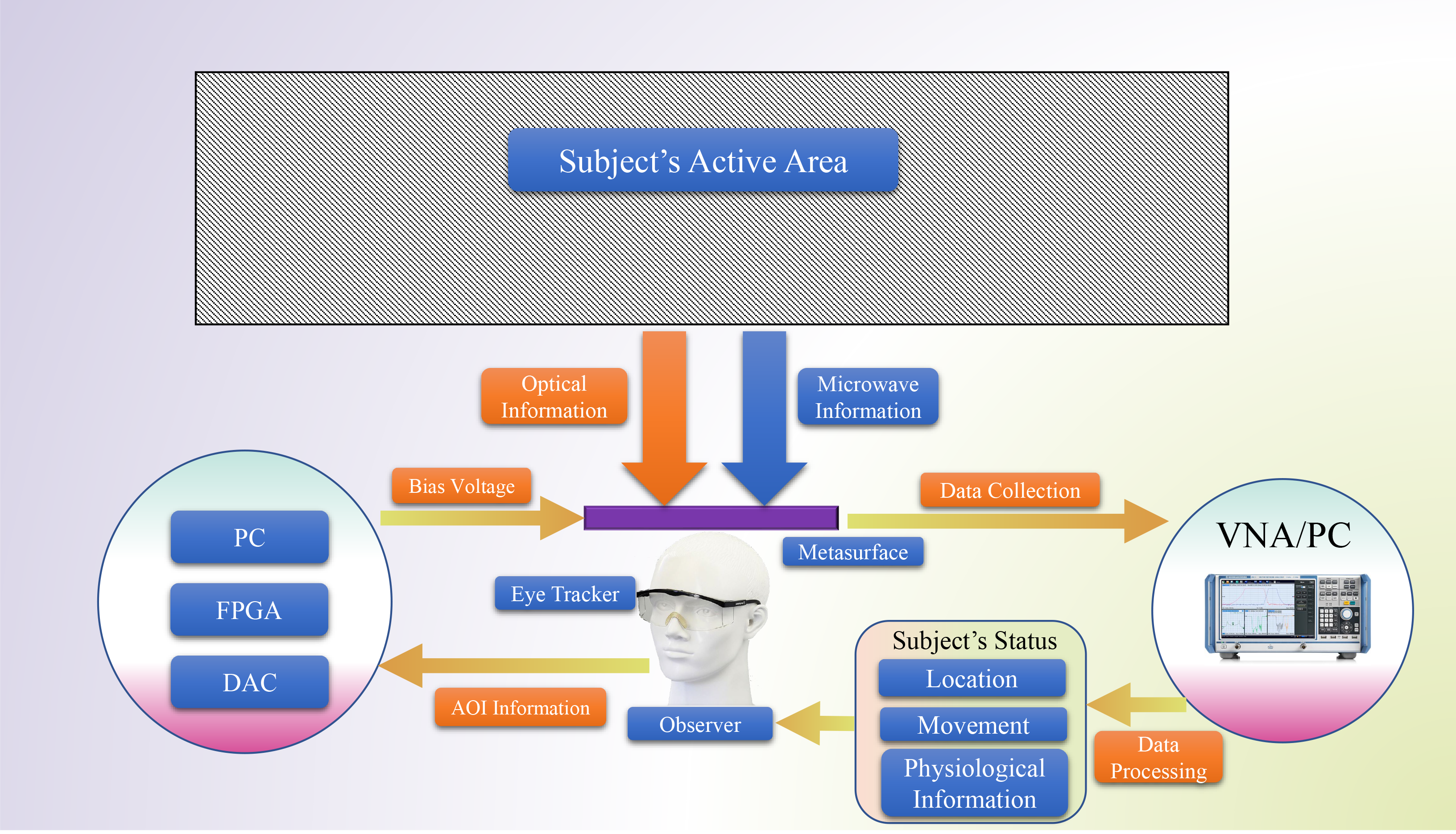


Fig. S7. The overall operation process flow chart of the experiment.

Fig. S7 illustrates the overall operation process of the experiment. The experimental process was performed in an outdoor stadium in order to imitate the application scenarios to the greatest extent. The observer with normal vision worn eye tracking glasses and observe the environment through the metasurface. A wearable display attaches to the eye tracker to expose observers to processed microwave information. EM waves emitted by the metasurface irradiated to the subjects, standard-gain antenna is used for receiving echo waves. The VNA was connected to the computer through the network cable to realize the real-time data collection. Other parts of the experiment were the same as the previous experiment in Supplementary Note S4.

The experiment was divided into three major parts: visible multitargets respiration and heartbeat signal detection, human location and motion detection behind plank obstacles, and establishment of human motion detection database behind plank obstacles.

1. **Visible multi-targets respiration and heartbeat signal detection**


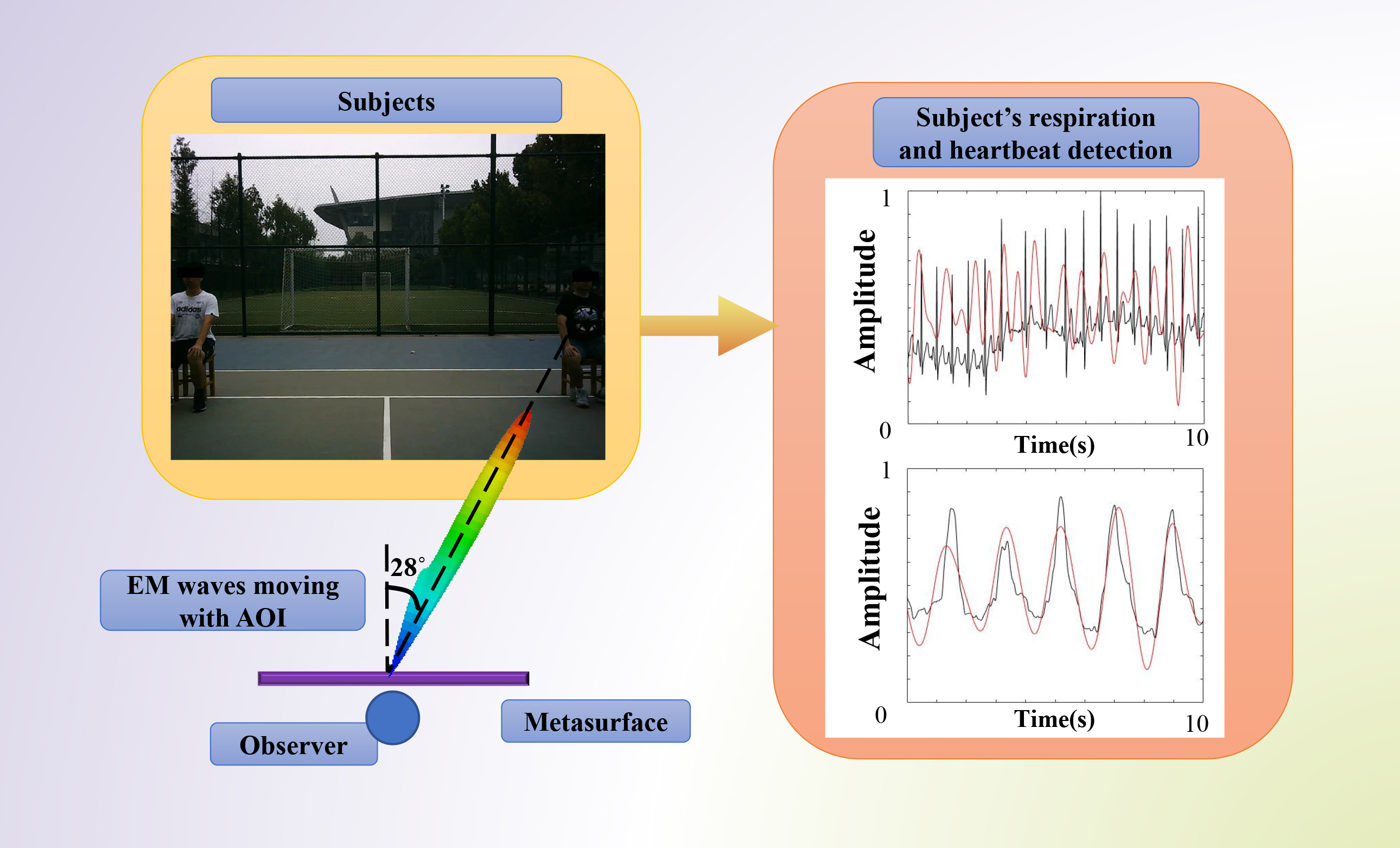


Fig. S8. The flowchart of visible multitargets respiration and heartbeat signal detection experimental setup.

As shown in Fig. S8, two subjects were asked to sit about 4 m away from the metasurface. The azimuth angle where the subjects are located is plus or minus 28 degrees. The observer fixated one of the subjects successively in each of the two sets of experiments in order to make EM waves irradiate the subjects. Two healthy adult subjects were asked to wear piezoelectric respiration belts (Chengyi RM6240e) and disposable ECG electrode connected to a portable electrocardiograph (iWorx IX-B3G) as the ground truth to verify the measured respiration and heartbeat signals of the system. To avoid the interference of random body movement, the persons under test were requested to sit steadily in resting state. Next, the reflection coefficient was collected by VNA, and then analyzed for respiration and heartbeat detection.

1. **Human location and motion detection behind plank obstacles**

Fig. S9 illustrates the experimental setup of human location and motion detection behind plank obstacles. In this experiment, plank obstacles were placed between observer and subject to block the path between them. For the sake of simplicity, three angles of the line connecting the subjects and the metasurface and the normal (-28˚, 0˚, 28˚) were selected in the experiment. One or three subjects was asked to stay in one of three specified angles behind plank obstacles. At this time, the observer was asked to fixate the three specified angles over the time threshold in sequence. And then the azimuth angle of the subject was determined in real time through analyzing reflection coefficient *S*_11_ collected by VNA and uploaded to the PC.


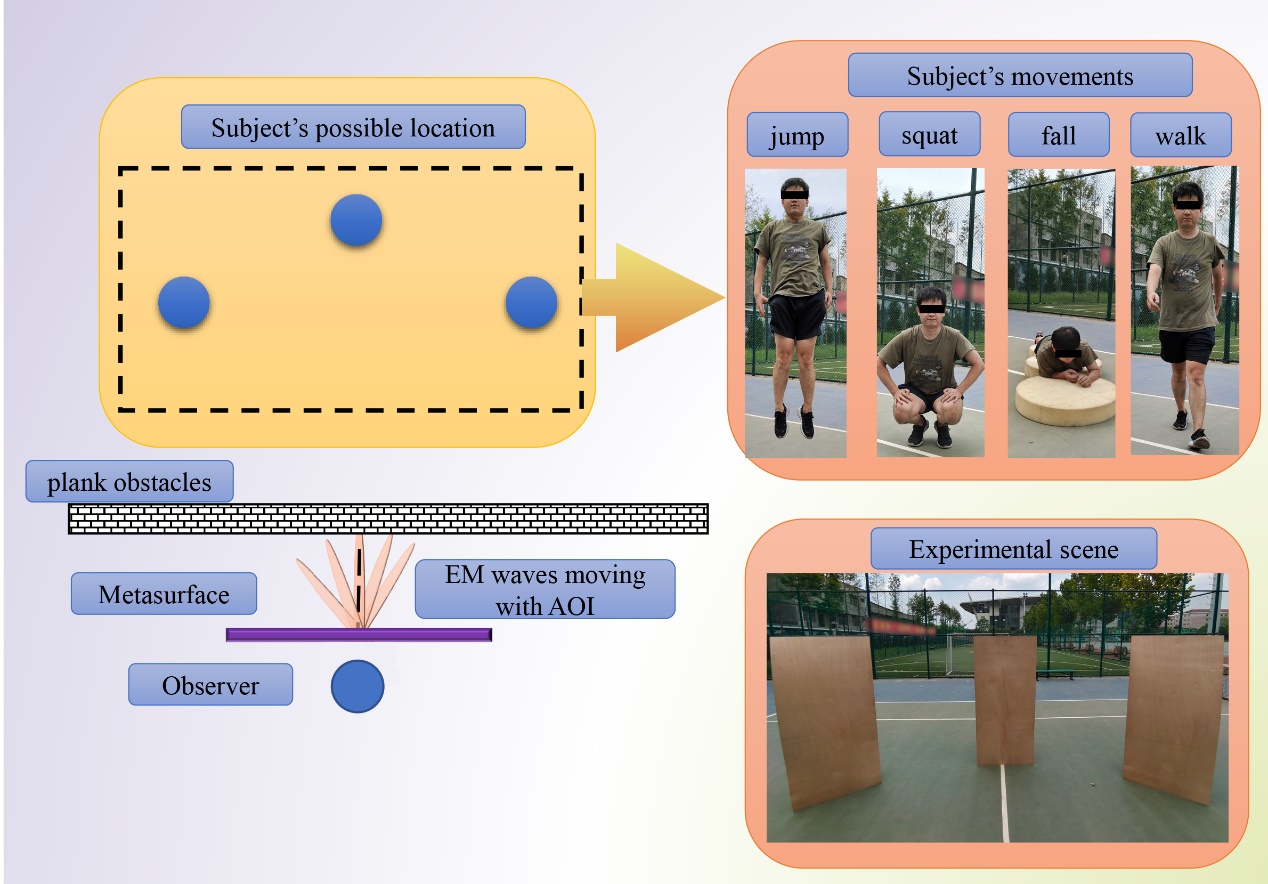


Fig. S9. The flowchart of human location and motion detection experimental setup.

Afterward, the subject began to perform one of the four movements, that is, jumping, squatting, falling, and walking at the specified azimuth. Each motion was recorded for a duration depending on the activity. Meanwhile, the observer was asked to fixate the azimuth angle where the subject locates over a period of time. Through analyzing reflection coefficient *S*_11_ collected by VNA, the trained model will automatically help the observer to determine the motion type of the blocked human subject.

**Supplementary Note S8: The experimental configuration for "glimpse-and-forget" metasurface smart target tracking system**


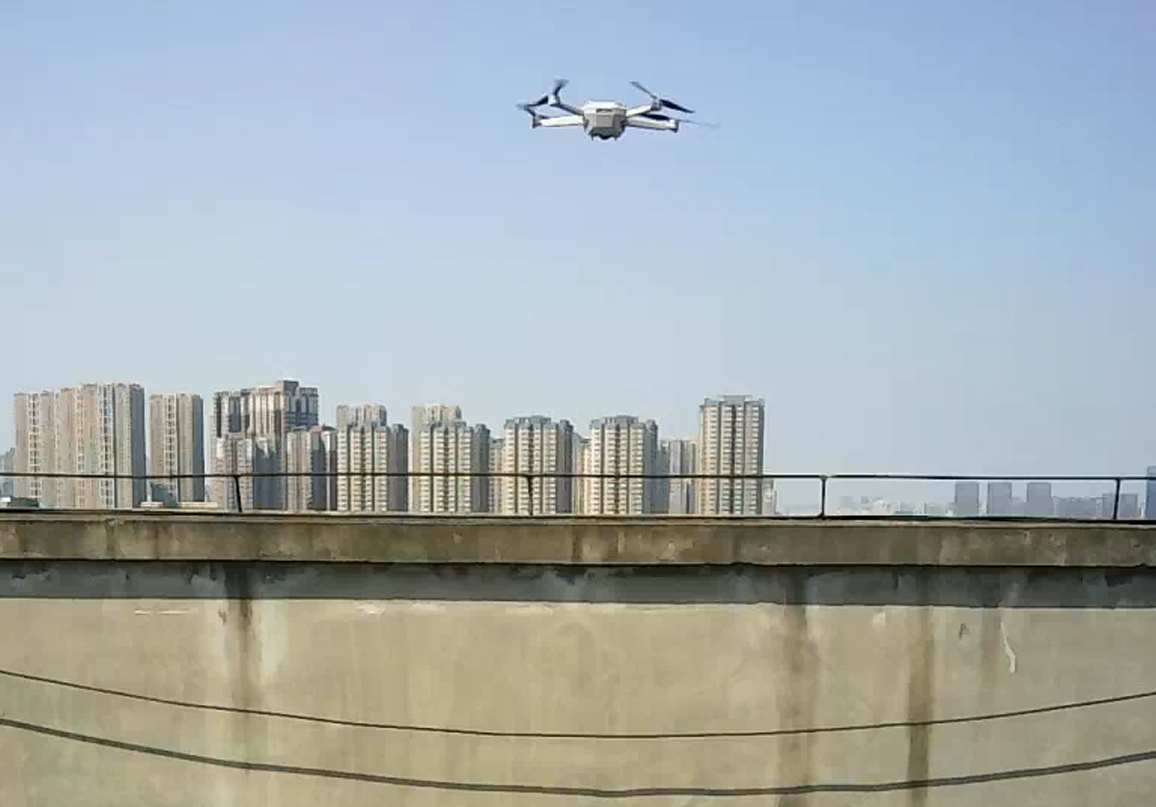


Fig. S10. The experimental scene photographs of the "glimpse-and-forget" metasurface smart target tracking system.

In the experiment, UAV (DJI mini 3 pro) was selected as track target. A variety of 3D movement trajectories were preset for the UAV, including movement in the horizontal and vertical plane. The experiment was carried out twice, the UAV flew in the horizontal plane for the first time, and flew in the vertical plane for the second time. The horizontal plane chosen was the plane on which the human eye is located. While the vertical plane chosen was the one 2.5 m from the metasurface. The metasurface emitted a 14.5 GHz CW signal with 0 dBm power. The UAV flew on the preset trajectory and the observer located the object to be tracked in the eye tracker through rapidly blinking their eyes. Other parts of the experiment were the same as the previous experiment in Supplementary Note S7.

**Supplementary Note S9: The principle of velocity measurement for "glimpse-and-forget" metasurface smart target tracking system**

According to Doppler effect, when the moving target approaches the metasurface system, the frequency of the echo signal becomes higher than the frequency of the transmission signal; When the target is away from the radar, the echo signal frequency decreases. The difference between the frequency of the transmitted signal and echo signal is defined as the Doppler frequency:

$f_{d}=\frac{2v_{r}}{\lambda}$  (S9)

where $v_{r}$ is the radial velocity of the target relative to metasurface, and *λ* is the wavelength of the emitted EM wave. It is generally agreed that *f_d_* is negative when the target is far from the metasurface and positive when the target is close to the metasurface. It can be seen that the radial velocity *v_r_* of the target can be obtained by the above formula if the Doppler frequency *f_d_* can be measured when the emission wavelength *λ* is known. It can also be seen that the accuracy of velocity measurement mainly depends on the detection accuracy of the Doppler frequency *f_d_* when the transmitting wavelength is fixed.

Suppose the CW signal emitted by the metasurface is:

$u_{t}(t)=A_{tm}\text{cos}\left( \omega_{c}t \right)$  (S10)

where *A_tm_* is the amplitude of the transmitted EM signal, and *ω_c_* is the angular frequency of the transmitted EM wave signal. The reflected echo signal by the target is denoted as:

$u_{r}(t)=A_{rm}\text{cos}\left( \omega_{r}t+\varphi\right)$  (S11)

where *A_rm_* is the amplitude of the echo signal, $\omega_{r}$ is the angular frequency of the echo signal, and $\varphi$ is the phase difference between the echo signal and the transmitted signal. By multiplying the echo signal and the transmitted signal, there arrives,

$\left. \left. A_{tm}\text{cos}\left( \omega_{c}t \right)\cdot A_{rm}\text{cos}\left( \omega_{r}t+\varphi\right)=\frac{A_{tm}\cdot A_{rm}}{2}\left\{ \left[ \text{cos}\left( \omega_{c}+\omega_{r} \right)t+\varphi\right) \right]+\left[ \text{cos}\left( \omega_{r}-\omega_{c} \right)t+\varphi\right) \right] \right\}$ (S12)

In the formula, the first term is the sum frequency component (high frequency component), which can be filtered by the LPF low-pass filter; The second term is the difference frequency component (low frequency component), which can be retained by the low-pass filter. This difference frequency component is also known as the IF signal.

The frequency of the IF signal is the Doppler frequency, which can be obtained by the FFT operation. Then the radial velocity of the target relative to the radar can be obtained by equation S9. On this basis, the flying velocity of UAV can be derived according to the geometric relationship between radial velocity and flying velocity.

**Supplementary Note S10: The experimental implementation details for "speech acquisition and enhancement system"**


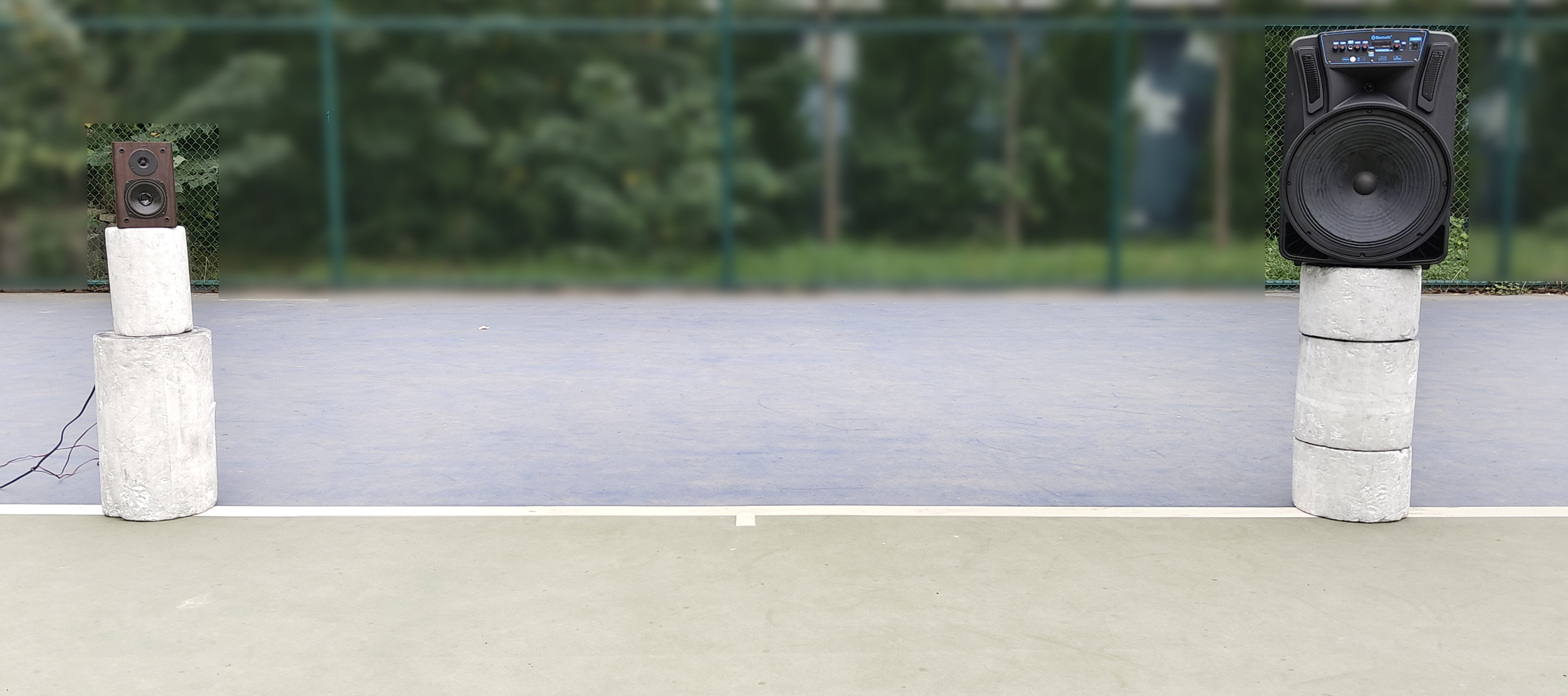


Fig. S11. The photographs of the experimental scene of "speech acquisition and enhancement system".

The speech detection experiment was performed in an outdoor stadium. The observer wore eye tracking glasses and a wearable display attached to the eye tracker is used to display the final processed microwave speech information. As shown in Fig. S11, two loudspeakers were placed about 4 m away from the metasurface with azimuth angles of plus and minus 28 degrees, respectively. The observer fixated one of the loudspeakers in order to make EM waves irradiate the loudspeaker. The loudspeaker fixated by the observer blared words with the content "Consider Support Fit Hang Protect Professional?" While the other loudspeaker blared babble noise which was defined as an overlay of at least 2 simultaneous speech sources. Babble noise is considered as one of the most suitable noises for masking speech. The metasurface emitted a 14.5 GHz CW signal with 20 dBm power. The recording lasted for 10 seconds, with a sample rate of 5000 Hz, which satisfied the Nyquist sampling requirement for audio signals. Other parts of the experiment were the same as the previous experiment in Supplementary Note S7.

**Supplementary Note S11: The theory and** **algorithm of speech acquisition and enhancement system**

The theory of speech signal acquisition is the same as that of vital sign detection, which has been detailed in Supplementary Note S5. For those who are interested, please kindly refer to that part for the details.

Because the raw extracted microwave speech information contains strong noise, which affects the accurate recognition of speech information. This work employs the widely utilized speech enhancement algorithm, namely minimum mean-square error based short-time spectral amplitude (MMSE-STSA), to suppress the noise.

Suppose that the authentical speech signal *x*(*t*) and the ambient noise signal *n*(*t*) are independent, which holds for this scenario, the following signal model can be obtained,

$y\left( t \right)=x\left( t \right)\boldsymbol{+}n(t)$ (S13)

where *y*(*t*) denotes the phased unwrapped noisy microwave speech signal.

Let $X_{k}=A_{k}\text{exp}(j\alpha_{k})$, $N_{k}$, and $Y_{k}=R_{k}\text{exp}(j{\alpha\theta}_{k})$ denote the *k*-th spectral component of the signal *x*(*t*), the noise *n*(*t*), and the noisy observation *y*(*t*), respectively. Also, the spectral components assume to follow the Gaussian statistical model.

Then, the MMSE STSA amplitude estimator can be written as below,

$\arg\min_{\hat{A_{k}}} E[\left( A_{k}-\hat{A_{k}} \right)^{2}|y(t), 0\leq t\leq T]$ (S14)

Our goal is to estimate *A_k_* from the noisy signal *y*(*t*). The optimal solution of equation S14 can be derived as,

$$\hat{A_{k}}=E\left[ A_{k} | y\left( t \right), 0\leq t\leq T \right]$$

$=\Gamma\left( 1,5 \right)\frac{\sqrt{v_{k}}}{\gamma_{k}}\exp\left( -\frac{v_{k}}{2} \right)\left[ \left( 1+v_{k} \right)I_{0}(\frac{v_{k}}{2})+v_{k}I_{1}(\frac{v_{k}}{2}) \right]R_{k}$ (S15)

Where *Γ* denotes the gamma function, *I_0_* and *I_1_* are modified Bessel function of zero and first order. *v_k_* is the gain value defined by prior SNR and posterior SNR *γ_k_*. Using this algorithm, the audio information can be well enhanced from the noisy microwave speech signal.
