# Supplementary material for: Vision-driven metasurfaces for perception enhancement
Source: Nat Commun. 2024 Feb 22;15:1631. doi: 10.1038/s41467-024-45296-x (PMC10883922; doi:10.1038/s41467-024-45296-x)
Supplement: Supplementary file 3 — Description of Additional Supplementary Files [file 41467_2024_45296_MOESM3_ESM.docx]

**Description of Additional Supplementary Files**

**Supplementary Audio 1:** The original voice audio file

**Supplementary Audio 2:** The voice audio file measured by metasurface after noise removal
