# Supplementary material for: Vision-driven metasurfaces for perception enhancement
Source: Nat Commun. 2024 Feb 22;15:1631. doi: 10.1038/s41467-024-45296-x (PMC10883922; doi:10.1038/s41467-024-45296-x)
Supplement: Supplementary file 6 — Reporting Summary [file 41467_2024_45296_MOESM6_ESM.pdf]

nature portfolio

Corresponding author(s): Jianqi Wang, Jiafu Wang, Cheng-Wei Qiu,Hao Lv, Shaobo Qu

Last updated by author(s): Dec 20, 2023

Reporting Summary

Nature Portfolio wishes to improve the reproducibility of the work that we publish. This form provides structure for consistency and transparency in reporting. For further information on Nature Portfolio policies, see our Editorial Policies and the Editorial Policy Checklist.

Statistics

For all statistical analyses, confirm that the following items are present in the figure legend, table legend, main text, or Methods section.

n/a	Confirmed	
☐	☒	The exact sample size (*n*) for each experimental group/condition, given as a discrete number and unit of measurement
☒	☐	A statement on whether measurements were taken from distinct samples or whether the same sample was measured repeatedly
☒	☐	The statistical test(s) used AND whether they are one- or two-sided<br>*Only common tests should be described solely by name; describe more complex techniques in the Methods section.*
☒	☐	A description of all covariates tested
☒	☐	A description of any assumptions or corrections, such as tests of normality and adjustment for multiple comparisons
☒	☐	A full description of the statistical parameters including central tendency (e.g. means) or other basic estimates (e.g. regression coefficient) AND variation (e.g. standard deviation) or associated estimates of uncertainty (e.g. confidence intervals)
☒	☐	For null hypothesis testing, the test statistic (e.g. *F*, *t*, *r*) with confidence intervals, effect sizes, degrees of freedom and *P* value noted<br>*Give P values as exact values whenever suitable.*
☒	☐	For Bayesian analysis, information on the choice of priors and Markov chain Monte Carlo settings
☒	☐	For hierarchical and complex designs, identification of the appropriate level for tests and full reporting of outcomes
☒	☐	Estimates of effect sizes (e.g. Cohen's *d*, Pearson's *r*), indicating how they were calculated

Our web collection on statistics for biologists contains articles on many of the points above.

Software and code

Policy information about availability of computer code

Data collection	The softwares used for data collection has been described in this paper (and its supplementary information files).
Data analysis	The softwares used for data analysis has been described in this paper(and its supplementary information files).

For manuscripts utilizing custom algorithms or software that are central to the research but not yet described in published literature, software must be made available to editors and reviewers. We strongly encourage code deposition in a community repository (e.g. GitHub). See the Nature Portfolio guidelines for submitting code & software for further information.

Data

Policy information about availability of data

All manuscripts must include a data availability statement. This statement should provide the following information, where applicable:

- Accession codes, unique identifiers, or web links for publicly available datasets
- A description of any restrictions on data availability
- For clinical datasets or third party data, please ensure that the statement adheres to our policy

All data generated or analysed during this study are included in this paper(and its supplementary information files).

Research involving human participants, their data, or biological material

Policy information about studies with human participants or human data. See also policy information about sex, gender (identity/presentation), and sexual orientation and race, ethnicity and racism.

Reporting on sex and gender	Sex and gender information of the participants was reported in Methods Section. The experiment aims to validate the perception enhancement capabilities of visually-driven metasurfaces and therefore does not involve the gender or sex analysis.
Reporting on race, ethnicity, or other socially relevant groupings	The experiment aims to validate the perception enhancement capabilities of visually-driven metasurfaces and therefore does not involve the race, ethnicity, or other socially relevant groupings analysis.
Population characteristics	The experiment aims to validate the perception enhancement capabilities of visually-driven metasurfaces and therefore does not involve population characteristics analysis.
Recruitment	Participants were recruited from the teachers and students at the university.
Ethics oversight	All experimental procedures involved were performed according to protocols approved by the Fourth Military Medical University. An informed consent was given by all participants.

Note that full information on the approval of the study protocol must also be provided in the manuscript.

Field-specific reporting

Please select the one below that is the best fit for your research. If you are not sure, read the appropriate sections before making your selection.

- [x] Life sciences
- [ ] Behavioural & social sciences
- [ ] Ecological, evolutionary & environmental sciences

For a reference copy of the document with all sections, see nature.com/documents/nr-reporting-summary-flat.pdf

Life sciences study design

All studies must disclose on these points even when the disclosure is negative.

Sample size	The sample size depends mainly on the configuration of the metasurface system. For example, the visible multi-targets' respiration and heartbeat detection section requires measuring the heartbeat of two participants.
Data exclusions	No data was excluded from the analyses.
Replication	All replication attempts were successful.
Randomization	All participants were allocated into experimental groups randomly.
Blinding	All the experiments were performed and analyzed in a blind manner.

Reporting for specific materials, systems and methods

We require information from authors about some types of materials, experimental systems and methods used in many studies. Here, indicate whether each material, system or method listed is relevant to your study. If you are not sure if a list item applies to your research, read the appropriate section before selecting a response.

Materials & experimental systems

n/a	Involved in the study	
x		Antibodies
x		Eukaryotic cell lines
x		Palaeontology and archaeology
x		Animals and other organisms
x		Clinical data
x		Dual use research of concern
x		Plants

Methods

n/a	Involved in the study	
x		ChIP-seq
x		Flow cytometry
x		MRI-based neuroimaging
